# An Innovative In Vitro Open-Angle Glaucoma Model (IVOM) Shows Changes Induced by Increased Ocular Pressure and Oxidative Stress

**DOI:** 10.3390/ijms222212129

**Published:** 2021-11-09

**Authors:** Stefania Vernazza, Sara Tirendi, Mario Passalacqua, Francesco Piacente, Sonia Scarfì, Francesco Oddone, Anna Maria Bassi

**Affiliations:** 1 Department of Experimental Medicine (DIMES), University of Genoa, 16132 Genoa, Italy; stefania.vernazza@yahoo.it (S.V.); tirendisara@gmail.com (S.T.); francesco.piacente@unige.it (F.P.); anna.maria.bassi@unige.it (A.M.B.); 2 Inter-University Center for the Promotion of the 3Rs Principles in Teaching & Research (Centro 3R), 56122 Pisa, Italy; soniascarfi@unige.it; 3 Department of Earth, Environmental and Life Sciences (DISTAV), University of Genoa, 16132 Genoa, Italy; 4 IRCCS-Fondazione Bietti, Via Livenza 3, 00198 Rome, Italy; francesco.oddone@fondazionebietti.it

**Keywords:** 3D model, glaucoma, trabecular meshwork, bioreactor

## Abstract

Primary Open-Angle Glaucoma (POAG) is a neurodegenerative disease, and its clinical outcomes lead to visual field constriction and blindness. POAG’s etiology is very complex and its pathogenesis is mainly explained through both mechanical and vascular theories. The trabecular meshwork (TM), the most sensitive tissue of the eye anterior segment to oxidative stress (OS), is the main tissue involved in early-stage POAG, characterized by an increase in pressure. Preclinical assessments of neuroprotective drugs on animal models have not always shown correspondence with human clinical studies. In addition, intra-ocular pressure management after a glaucoma diagnosis does not always prevent blindness. Recently, we have been developing an innovative in vitro 3Dadvanced human trabecular cell model on a millifluidicplatform as a tool to improve glaucoma studies. Herein, we analyze the effects of prolonged increased pressure alone and, in association with OS, on such in vitro platform. Moreover, we verify whethersuch damaged TM triggers apoptosis on neuron-like cells. The preliminary results show that TM cells are less sensitive to pressure elevation than OS, and OS-damaging effects were worsened by the pressure increase. The stressed TM releases harmful signals, which increase apoptosis stimuli on neuron-like cells, suggesting its pivotal role in the glaucoma cascade.

## 1. Introduction

Glaucoma comprises a group of progressive neurodegenerative eye diseases characterized by a selective loss of retinal ganglion cells (RGCs) and their axons, the cupping of the optic nerve head, as well as the loss of vision [1,2]. 

Primary open-angle glaucoma (POAG), the most common form of glaucoma (about 70%), is anatomically characterized by an open-iridocorneal angle, as well as (but not always) by an increase in the intraocular pressure (IOP) [3]. Therefore, the presence or absence of an increase in the IOP enables a distinction to be made between High-Tension Glaucoma (HTG) and Normal-Tension Glaucoma (NTG) [4]. 

Although in HTG, both the progressive morphofunctional decay of trabecular meshwork (TM) (e.g., TM stiffening) and the reduction in its cell number are responsible for IOP elevation [5,6], it is reasonable to ask whether such changes are also responsible for the molecular signals thatare capable of triggering RGC apoptosis [7]. 

Currently, glaucoma therapy is mainly focused on lowering the IOP, with the aim ofslowing down disease progression. However, this therapeutic approach, in some cases, does not necessarily stop RGC loss, and can still lead to progressive blindness [1,8]. Hence, the explanation for why lowering the IOP does not always provide a satisfactory outcome may well be related to a variety of molecular signals involved in glaucoma pathogenesis, including oxidative stress, mitochondrial damage, apoptotic signals arising from different ocular areas, glutamate excitotoxicity, pro-inflammatory cytokines and the disruption of the integrity of the blood–retinal barrier [9,10]. 

In this regard, for several years, neuroprotective strategies thatare able to block RGC damage in glaucoma [11,12,13,14,15,16] have been investigated and developed. However, whilst the results obtained from experiments carried out on small animals, related to the effectiveness of neuroprotective medicines, are promising, confirmation has not always been found in clinical trials. Moreover, it must be highlighted that the use of animal models, such as non-human primates, which anatomically are more similar to humans, is constrained by both ethical and regulatory issues. 

Therefore, there is the clinical need to develop additional therapeutic strategies [17], starting from in vitro human-based platforms, in order to enhance an accurate and predictive drug testing process regardless (at least partially) of the animal used. 

Although animal models have proven useful for some aspects of our current knowledge on glaucoma, there is growing skepticism about their contribution to our understanding of the onset of glaucoma and its propagations, as well as its treatment in humans [18]. 

The improvement of three-dimensional (3D) cultures for cell-based experiments could represent the key to understanding the biology of glaucoma in more detail, thus resulting in better and more effective conditions in which to identify the initial molecular events thatlead to changes in physiological conditions in an attempt to discover new treatment strategies. Furthermore, advanced glaucoma screening models can decrease the required time and costs of drug development in terms of both the reduction in the number of animals used during pre-clinical phases [19] and the money invested by pharmaceutical companies during the clinical trial phases. 

The possibility of isolating cells from human living tissues and culturing them in 3D in vitro models means that the two-dimensional in vitro system’s limitations can be overcome due to the fact that optimal spatial cell organization, in which cell–cell and cell–matrix interactions are maintained, closely resembles the natural shape and the biological response of in vivo conditions [20]. Indeed, over recent years, a wide range of 3D cell culture advanced platforms have been proposed (e.g., multicellular spheroid formation, hydrogel-based cultures, bioreactor-based cultures, bio-printing and scaffold-based cultures) [20,21], and all these techniques/platforms have the same aim of providing morphological, functional, and micro-environmental featuressimilar to those found in vivo. 

More recently, in vitro approaches toglaucoma research have been improved by hydrogel-based 3D human trabecular meshwork (h-TM) models that consist of cross-linked networks of natural polymeric biomaterials in which the cells are embedded [22,23]. These hydrogel techniques resemble an ideal environment in which to mimic the native cell’s architecture and molecular crosstalk, as well as providing anatural extra-cellular matrix (ECM), since they are combined with a reconstituted basement membrane preparation extracted from mouse sarcoma, namely, Matrigel^TM^.

Additionally, 3D h-TM models have been obtained using pre-customized 3D scaffolds based on either collagen–chondroitin sulfate [24] or synthetic SU-8 scaffolds [25]. Once again, these porous scaffolds are biocompatible and mimic the in vivo ECM, resulting in an appropriate cell microenvironment.

However, 3D culture models can only succeed if they are utilized together with knowledge from the science of materials, cell biology, bioreactor systems and so on.

We have already reported evidence on the improvement of the 3D h-TM models’performance by applying millifluidic technology using bioreactor systems. During the exposure to prolonged oxidative stress, this dynamic platform showed the more realistic behavior of 3D h-TM cells compared to 3D h-TM, cultured under static conditions, in terms of maintaining cell survival and triggering adaptive mechanisms towards stressors [26]. Indeed, the application of the millifluidic compartmentalization turned out to be a useful tool in examining both the h-TM cell behavior after exposure to hydrogen peroxide, as an oxidative stressor, and in enabling the analysis of the complex interactions between h-TM cells and their micro environmental components through an accurate and reproducible model. In fact, the limits of 3Dstatic systems are mainly related to a lack of oxygen availability [27], whereas the dynamic millifluidic platform provides a constant flow rate of the culture medium within the whole bioreactor circuit, allowing a continuous nutrient supply of oxygen to the connected multi-culture chambers, and thus overcoming not only the mass transport limitations, but also removing the metabolic products from the cells.

Furthermore, recently, we have demonstrated that our 3Dadvanced model of h-TM can also be used for screening studies involving antioxidant compounds of novel agents to restore TM functionality after oxidative stress (OS) damage [28]. 

Although little is known about the pathological role of the damaged TM in glaucoma on surrounding tissues, and in particular on neural cells, there is evidence for an increase in TM stiffness and IOP elevation. However, it is believed that the TM-derived molecules from damaged TM can also compromise RGC homeostasis in acting as pro-apoptotic signals [7]. 

The aims of the present study were firstly to evaluate the effects of oxidative stress and increased pressure alone, and their association, on the dynamic in vitro 3D h-TM cell model. Secondly, to improve our knowledge on the influence of stressed TM cells on human neuron-like cells, retinoic acid (RA)-induced differentiated human neuroblastoma SH-SY5Y cells (RA-SH-SY5Y) were grown in the presence of experimental TM-conditioned media, using the bioreactor platform.

To our knowledge, this is the first in vitro approach based on human cells cultured on a millifluidic platform, applying a device as a mechanical stressor to modulate medium flow pressure, with or without OS, or to simulate the two known pathological conditions that are present in glaucoma.

## 2. Results 

**Figure 1:**
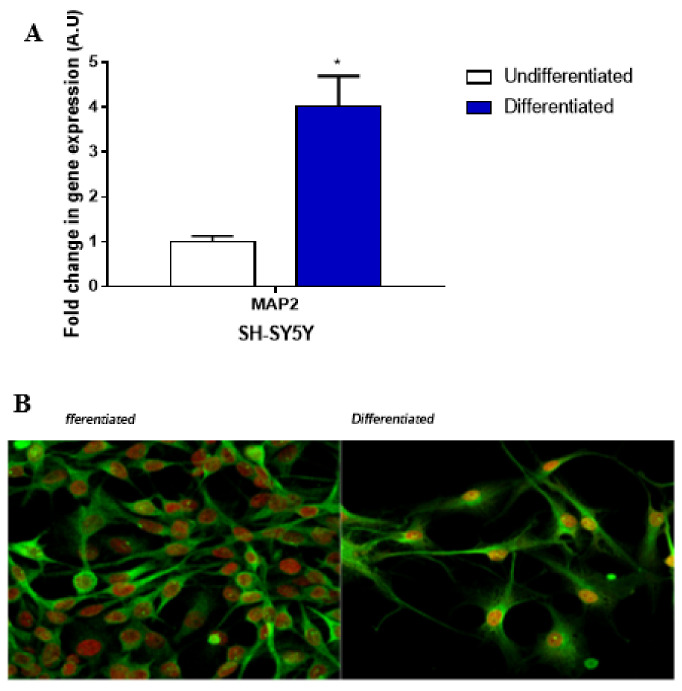
SH-SY5Y differentiation. (**A**) RT-qPCR analysis of MAP2 gene normalized to relative HPRT levels in both undifferentiated SH-SY5Yand RA-SH-SY5Y cells. * *p* < 0.05 vs. undifferentiated SH-SY5Y. (**B**) SH-SY5Y and RA-SH-SY5Y cells immunostained for βIII-Tubulin antibody (neuronal marker; green) and To-Pro (nuclear marker; blue).

In order to verify RA-SH-SY5Y differentiation, the gene expression of MAP2, a primary feature of neuronal differentiated cells required for dendrite elongation, and β III tubulin positive-immunofluorescent staining were analyzed (Figure 1). As regardsthe MAP2 gene expression levels, a 5-fold increase in RA-SH-SY5Ycells compared to undifferentiated ones was observed. The neuronal phenotype of RA-SH-SY5Ycells was further confirmed by labeling cells with an antibody for neuron-specific βIII tubulin, which is considered a marker of neurogenesis, axon guidance and maintenance. RA-SH-SY5Ycells displayed a positive labelling forβ-III tubulin, showing an outgrowth of neurites and a decrease in the rate of cell proliferation compared to the undifferentiated SH-SY5Y cells. 

### 2.1. Viability

**Figure 2:**
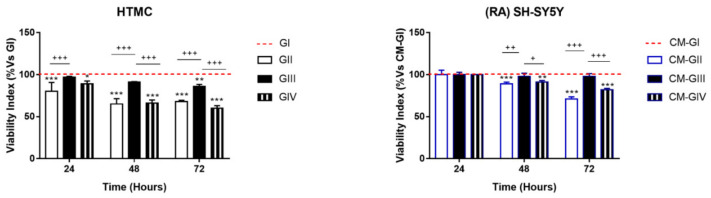
Alamar Blue assay. During experimental treatments, the metabolic state of HTMC and RA-SH-SY5Ywas evaluated by Alamar Blue assay. The bar diagrams show the viability indices in terms of resazurin reduction and are expressed as % of their respective control cell group. Each bar represents the mean ± S.D. of 3 separated experiments in triplicate. *** *p* < 0.001, ** *p* < 0.01, * *p* < 0.05 vs. control cell group; +++ *p* < 0.001, ++ *p* < 0.01, + *p* < 0.05 vs. treated cell groups (Two-way ANOVA followed by Bonferroni posttest).

The Alamar Blue assay is considered a healthycell marker, which monitors metabolicallyactive cells and provides a quantitative analysis of the cell viability and proliferation (Figure 2). HTMCs showed a significant reduction (20%) of Alamar Blue index after just 24 h of GII and GIV experimental conditions, which were associated with the direct exposure to daily doses of hydrogen peroxide, whilst in the other experimental conditions, in which HTMCs were subjected only to PE (i.e., GIII), a significant reduction of about 4% was reported at 72 h.

Moreover, RA-SH-SY5Y cells grown in CM from HTMC treated under GII and GIV experimental conditions evidenced delayed damage, since a significant viability reduction was only observed at 48 h onwards.

### 2.2. Apoptosis

**Figure 3:**
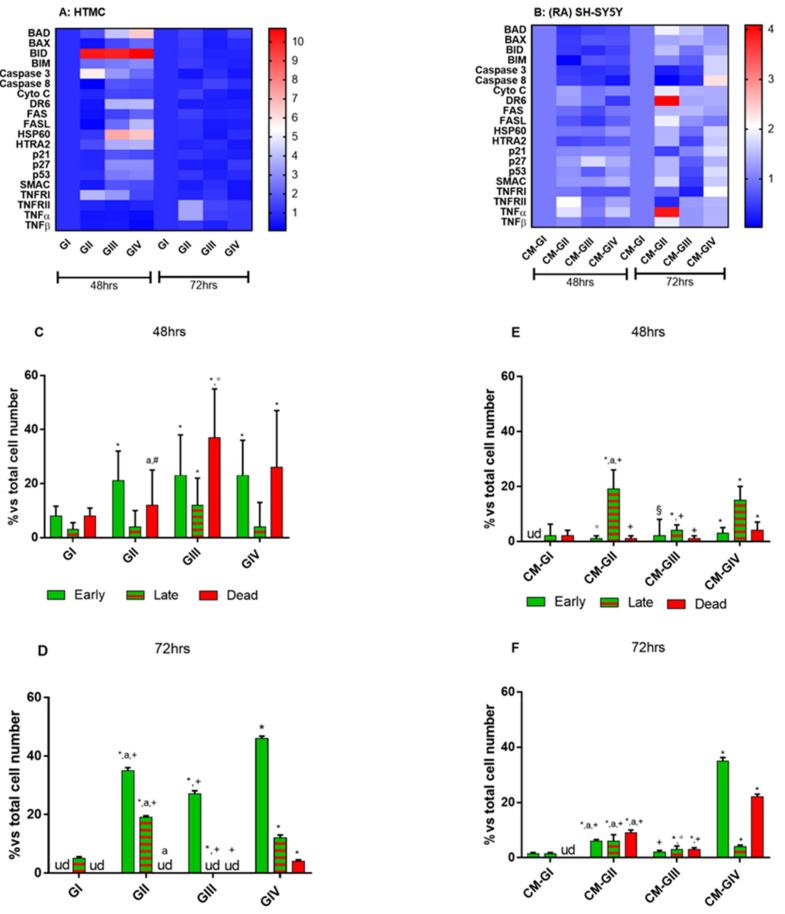
Apoptosis pathway analysis. Heat map of pro-apoptotic protein levels analyzed both in HTMCs (**A**) and RA-SH-SY5Ys (**B**). The color scale represents the value relating to pro-apoptotic protein levels in the various treatment groups. An Annexin V-FITC/PI-stained fluorescence microscope was used to evaluate the different stages of apoptosis in HTMC (**C**,**D**) and in RA-SH-SY5Y (**E**,**F**). The bar diagram shows early (green bars) and late (green/red bars) apoptosis and death cells (red bars) after 48 and 72 h of experimental procedures. * *p* < 0.001 vs. control cell group; a, #, +, ° *p* < 0.001; § *p* < 0.01 vs. treated cell group (Two-way ANOVA followed by Bonferroni posttest).

The apoptosis pathway in HTMCs and RA-SH-SY5Ys, treated as mentioned, was verified by a commercial apoptosis protein array, which allows for the analysis of multiple pro-apoptotic proteins simultaneously (Figure 3A,B). Moreover, in order to distinguish the different labeling patterns of the apoptosis steps within the cell populations, a fluorescent Annexin V-FITC/PI kit stain kit was used (Figure 3C–F). 

### 2.3. Apoptosis Array

The heat map visualization of the differentiallyexpressed pro-apoptotic proteins reveals an upregulation of seventeen apoptosis-related proteins in HTMCs, prevalently at 48 h, under both GIII and GIV conditions, whilst under GII conditions, only seven had upregulated their expressions (Figure 3A). In particular, the HTMCs, included under GIII and GIV conditions, showed an increase in BID, a pro-apoptotic protein that drives apoptosis via the mitochondria pathway, as well as of HSP60, a molecular chaperon mainly located in the mitochondria [29]. Conversely, RA-SH-SY5Ys, underto CM-GII and CM-GIV conditions, upregulated eight apoptotic proteins (*p* < 0.01), each being different from one another, after only 72 h of experimental procedures, compared to both CM-GI and CM-GIII. No statisticallyremarkable changes between CM-GI and CM-GIII were observed (Figure 3B). 

### 2.4. Annexin V-FITC/PI Stained Fluorescence Microscopy

HTMCs and RA-SH-SY5Ys stained with Annexin V-FITC/PI revealed a percentage of early apoptotic cells (Annexin V-reactive cells), late apoptotic/necrotic cells (Annexin V- and PI-reactive cells) and dead cells (PI-reactive cells) after experimental treatments. In HTMCs, at 48 h (Figure 3C), the percentage of early apoptotic cells increased by about 13% in all experimental groups, whilst the percentage of apoptotic/necrotic cells increased by about 7% in only GIII compared to the untreated group. Even though the increase in dead cells was significant underall experimental conditions, it was only under GIII conditions that they increased by 37% compared to the untreated cells. However, at 72 h, an increase in early apoptotic cells was observed in all HTMC experimental groups (Figure 3D), whilst apoptotic/necrotic cells were found in GII and GIV and dead cells only in GIV. 

The apoptotic trend in RA-SH-SY5Y, in contrast with the apoptosis array results, as early as 48 h revealed a significant percentage increase in both early apoptotic and apoptotic/necrotic cells underall experimental conditions, whilst dead cells were only found under CM-GIV conditions (Figure 3E). However, at 72 h, the results confirmed that the combined effects of oxidative stress and pressure elevation indirectly enhanced the death of RA-SH-SY5Ys compared to the single effect of each stimulus (Figure 3F). 

### 2.5. Mitochondrial Function 

Mitochondria are essential organelles with a critical role in different cellular processes, ranging from energy production to apoptosis. As is well known, mitochondria dysfunction is recognized as one of the common pathological pathways in glaucoma, not only because it results in an increase inROS production, but because under such conditions of elevated ROS, a rapid depolarization of the inner mitochondria membrane potential and an impairment of the respiratory chain occurs. Therefore, it is not surprising that mitochondrial damage impacts cellular death in several ways [30]. 

**Figure 4:**
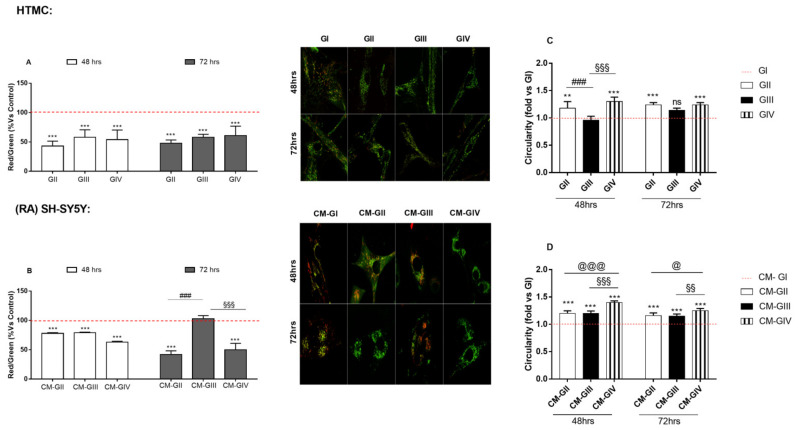
Mitochondrial membrane potential analysis. (**A**,**B**). The mitochondrial transmembrane potential was monitored in all experimental conditions by the JC-1 fluorochrome. On the left, quantitative analysis of ratio of red/green fluorescent intensity. Both red and green intensity were calculated for atleast 3 images of the same condition. Each measurement included ten areas of interest per image. On the right, confocal microscopy analyses of mitochondrial membrane potential were performed on 3D HTMC. Merged images show JC-1 dye both as monomer (green) and as J-aggregates (red). Mitochondrial morphology (**C**,**D**). The mitochondrial morphology was evaluated by MitoTracker analysis. The bar diagram shows mitochondrial morphological changes both in HTMC and RA-SH-SY5Y analyzed by means of circularity. *** *p* < 0.001; ** *p* < 0.01 vs. control cell group; ###, §§§, @@@ *p* < 0.001; §§ *p* < 0.01; @ *p* < 0.05 vs. treated cell group (Two-way ANOVA followed by Bonferroni posttest).

For the experiments, firstly, the mitochondrial activity was analyzed via JC1. As expected, and the HTMCs under GII, GIII and GIV conditions showed significantly reduced fluorescence ratios compared to GI, suggesting that mitochondria activity was altered under all experimental conditions (Figure 4A). The loss in the mitochondria membrane potential, which indicates the onset of apoptosis, could be considered a more likely and more accurate signal of apoptosis than caspase pathway activation or phosphatidylserine exposure. However, mitochondrial activity in RA-SH-SY5Y (Figure 4B) was significantly affected at 48 h by all treated HTMC-derived media, but this impairment continued to worsen at only 72 h under experimental conditions, in which the HTMC were previously exposed to either chronic oxidative stress or both stimuli (i.e., GII and GIV).

Secondly, the mitochondrial swelling was analyzed by the MitoTracker probe. The results indicate that only chronic oxidative stress and the combined effects of oxidative stress and pressure fluctuations induced alterations in the internal mitochondrial membrane of HTMC (Figure 4C). Conversely, RA-SH-SY5Y showed mitochondrial swelling under all treated HTMC-derived media conditions (i.e., CM-GII, CM-GIII and CM-GIV) starting from 48 h (Figure 4D), although CM-GIII did not alter the mitochondrialactivity alteration at 72 h. 

## 3. Discussion

This study reports a novel in vitro approach used to deepen the knowledge of both TM cell behavior under chronic oxidative/mechanical stimuli and the pathological contribution of TM cells totriggering neuron-like cell apoptosis. For the first time, we describe here an advanced in vitro model thattakes into consideration the early biological responses of two of the main tissues involved in POAG. 

This model shows that oxidative stress (OS) plays a pivotal role in HTMC degeneration, compared to the increase inpressure alone. In fact, on the basis of the results obtained by apoptosis array, JC1 and MitoTracker, both HTMC and RA-SH-SY5Y are less sensitive to pressure elevation than OS or OS combined with pressure elevation. Interestingly, when both stimuli were merged, as in the case of an advanced glaucoma stage, OS-related damage was exacerbated by the pressure increase in HTMCs which, in turn, affected RA-SH-SY5Ycell behavior. Therefore, the early OS-related mitochondria damage promotes further oxidative damage, not just of HTMC cells, but also of RA-SH-SY5Y cells. As is well known, mitochondria are important organelles involved in different cellular processes, including oxidative phosphorilation, the regulation of ROS production, and apoptosis [31]. However, their dysfunction contributes to neurodegenerative processes, such as amyotrophic lateral sclerosis, Alzheimer’s disease, Parkinson’s disease and glaucoma [32]. 

Although clinical treatments attempt to lower the IOP to treat all glaucoma forms, many patients continue to lose their eyesight, suggesting that other mechanisms may also be involved [33,34]. Moreover, given the complexity of glaucoma, the intraocular pressure fluctuations in themselves do not necessarily impact the optic projection in the same way in every individual (e.g., NTG) [35]. This present study aims to provide a new reading of TM damage involvement as a possible promoter of the RGC dysfunctional state, and not only as being responsible for an increase in the IOP. In general, since little is known about the molecular and cellular mechanisms underlying RGC damage, we can assume, at least in some cases, that there might be a cellular cross-talk between the TM and the RGCs. 

Our results demonstrate that treated HTMCs release signals or substances into medium thatare toxic to the RA-SH-SY5Ys, leading us to assume that, in glaucoma, RGC cell death could also be promoted by the pro-apoptotic signals of damaged TM [7]. Moreover, even though OS-treated HTMCs tended to reduce the apoptosis markerswithin 72 h, the mitochondrial damage thatpersisted actually increased the apoptosis of RA-SH-SY5Y over time.

However, this study can be considered as a preliminary approach to understand the role of TM in the glaucomatous cascade. Indeed, this in vitro platform will be improved by using human RGCs differentiated from Induced Pluripotent Stem Cells (iPSCs). As such, it will be possible to confirm and to characterize the pathological cross-talk between damaged TM cells and RGCs.

## 4. Materials and Methods 

### 4.1. Cell Cultures

The human trabecular meshwork cells (HTMC) and Trabecular Meshwork Growth Medium (TMGM) came from Cell APPLICATION INC. (San Diego, CA, USA). The cells were characterized according to Keller et al.’s [36] recommendations. The HTMC growth phase was supported by TMGM while, under experimental conditions, HTMCs were cultured with Dulbecco’s modified eagle’s medium (DMEM) containing a 1:1 mix of low and high glucose, 2 mM L-glutamine, antibiotics (0.5% gentamicin) and streptomycin (100 μg/mL), w/o fetal bovine serum. When the original flask was approximately 75% confluent, HTMCs were subculturedwithTrypLE™ Express Enzyme (Invitrogen Life Technologies, Carlsbad, CA, USA).

Human neuroblastoma cells (SH-SY5Y) from the American Type Culture Collection (Gaithersburg, MD, USA) were cultured in RPMI 1640 medium (Euroclone s.p.a., Milano, Italy) supplemented with 10% fetal bovine serum (Euroclone), 2 mM glutamine (Sigma-Aldrich, Darmstadt, Germany), 2% non-essential amino acids (Euroclone), 1% sodium pyruvate (Euroclone) and 1% penicillin/streptomycin (Euroclone). SH-SY5Ys underwent differentiation towards a normal phenotype following 10 μM retinoic acid (RA) treatment for 7 days, according to Nitti et al. [37]. The acquired neuronal phenotype of RA-SH-SY5Y cells was monitored by checking the gene expression of a neuronal polarity marker, i.e., MAP2 (Microtubule-Associated Protein 2) and the positivity of neuronal class β-III-tubulin using confocal microscopy images. 

All cell cultures were found to be mycoplasma-free during regular checks with the Reagent Set Mycoplasma Euroclone (Euroclone). 

### 4.2. qPCR

MAP2 gene expression, as a marker of the differentiation of RA-SH-SY5Y towards the neurogenic lineage, was evaluated by qPCR analysis and compared to undifferentiated SH-SY5Y. Total RNA was extracted using the RNeasy Micro Kit (Quiagen, Milano, Italy) according to the manufacturer’s instructions. RNA was quantified using aNanoDrop spectrophotometer (NanoDrop Technologies, Wilmington, DE, USA) and 150 ng per sample of cDNA was synthesized using the SuperScript^TM^ III First Strand Synthesis System (ThermoFisher Scientific, Monza, Italy). Each PCR reaction was performed as described elsewhere [23]. The values have beennormalized to HPRT. Primers were designed using the Beacon Designer 7.0 software (Premier Biosoft International, Palo Alto, CA, USA) and obtained from TibMolBiol (Genoa, Italy) (Table 1). Data analyses were obtained using the DNA Engine Opticon^®^ 3 Real-Time Detection System Software program (3.03 version) and, in order to calculate the relative gene expression of MAP2 compared to undifferentiated cells, the comparative threshold Ct method was used within the Gene Expression Analysis for iCycleriQ Real-Time Detection System software (Bio-Rad, Milano, Italy). 

### 4.3. Dynamic 3D Culture Conditions 

After 24 h of seeding, both 3D-HTMC and RA-SH-SY5Y underwent bioreactor analysis, usinga sophisticated model of a milli-scaled multi-organ device in a single-flow configuration (LB1, IVTechsrl, Massarosa, Lucca, Italy), equipped with a peristaltic pump (LF, IVTechsrl), transparent culture chambers, and a mixing bottle. The diagram of the bioreactor circuit, whichtechnology allows for the generation and precise tuning of the dynamic flow in order to deliver nutrients in a controlled and constant manner, has already been givenin our previous work [26]. 

**Figure 5:**
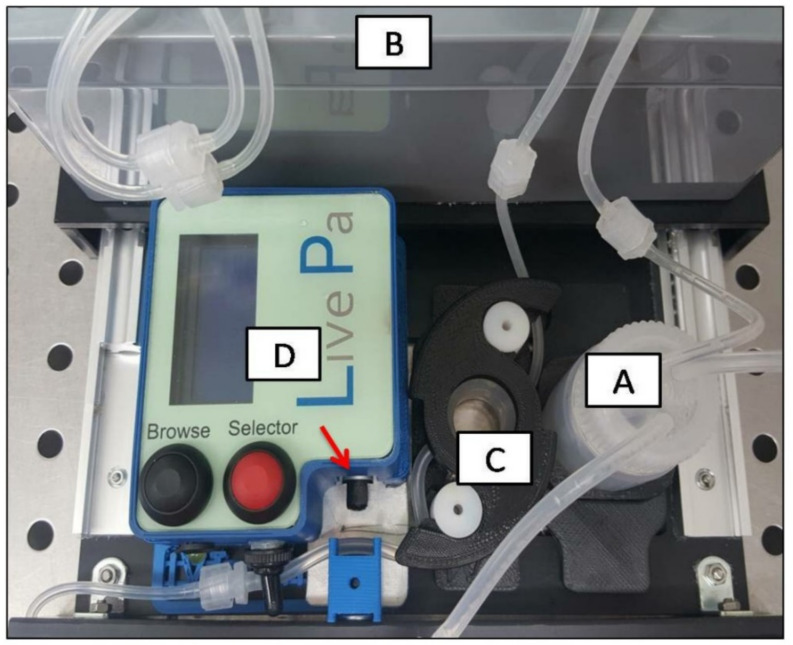
Perfusion bioreactor circuit diagram with the addition of Live Pa to increase culture chamber pressure. From the mixing bottle(**A**), the medium was pumped by the action of a peristaltic pump (**B**) through the perfusion chamber where cells had been seeded (**C**). The Live Pa (**D**) was designed to press the outlet tube of the perfusion chamber (red arrow) in order to increase the pressure within the perfusion chamber. Then, the medium returned to the mixing bottle, completing the circuit. (The figure has been kindly provided by IVTechsrl).

Moreover, to mimic the increase inpressure in the culture chamber (i.e., the pressure elevation condition), the in vitro platform was equipped with an auxiliary device consisting of a small plunger that, by pressing the outlet pipe of LB1, increased the base-line pressure by 10% (from 0.014 KPa to 0.021 KPa). The pressure oscillations between basal and elevated pressure followed the circadian rhythm (Figure 5). 

To assess the separated andcombined effects of OS and pressure elevation (PE), the experimental design employedfour experimental treatment conditions of the 3DHTMCs, as follows:

Group I (untreated control, UT)—3DHTMCs cultured in the presence of HTMC medium w/o FBS (see *Cell Cultures* section) and maintained under dynamic conditions for 72 h.

Group II (OS condition)—3DHTMCs exposed to 500 μM H_2_O_2_ for 2 h/day under static conditions and later subjected to dynamic conditions for 22 h/day up to 72 h [38,39].

Group III (PE condition)—3DHTMCs subjected to pressure fluctuations from base-line pressure (BP) within the culture chamber to a 10% pressure increase, following the circadian rhythm, which means 12 h/day of BP and 12 h/day of PE up to 72 h.

Group IV (OS+PE conditions)—3DHTMCs subjected contemporarily to OS and PE conditions (as described above) for up to 72 h.

### 4.4. RA-SH-SY5Y Experimental Design: Effects of the HTMCConditioned Medium 

In order to investigate whether, during experimental treatment, 3DHTMCs can release biomolecules that are able to induce direct cytotoxic effects on RA-SH-SY5Y cells, at the end of each experimental time, the conditionedmedium (CM) of each 3DHTMC experimental group (CM-Gs) was collected, centrifuged at 90 rcf and filtered through a 0.22 μm filter. CM-Gs were used as freshlycollected or stored at −20 °C until required for use (but not for morethan 1 month). 

RA-SH-SY5Ycells were exposed to CM-GI, CM-GII, CM-GIII, and CM-GIV under dynamic conditions. Moreover, the RA-SH-SY5Y cells, treated with CM-GIII and CM-GIV, were also subjected to pressure fluctuations (from BP to PE) up to 72 h. 

### 4.5. Viability 

Alamar Blue assay (Invitrogen^TM^, Life Technologies, Carlsbad, CA, USA) was performed daily to analyze the metabolic activity of 3DHTMC and RA-SH-SY5Y cells, according to the manufacturer’s protocol. Briefly, 10% (*v*/*v*) of Alamar Blue solution was added to each culture chamber and, after 4 hof incubation, the resazurin reduction was quantified spectrophotometrically at wavelengths of 570 and 630 nm. 

### 4.6. Mitochondrial Transmembrane Potential Analysis with the JC-1 Fluorochrome

The mitochondrial transmembrane potential of both 3DHTMC and RA-SH-SY5Y cells was analyzed by JC-1, a fluorescent dye localized exclusively in the mitochondria of living cells. At low concentrations, it exists as a monomer and emits green fluorescence, while at higher concentrations, it forms J-aggregates and emits red fluorescence. Therefore, in observing the red-to-green JC-1 fluorescence ratio, it is possible to extrapolate the membrane potential of mitochondria. 

A stock solution (5 mg/mL) of JC-1 (Thermo Fisher Scientific) was dissolved in dimethyl sulfoxide (DMSO) (Sigma-Aldrich) and stored at −20 °C. A fresh staining solution (2.5 μg/mL) was prepared each time before use by diluting the stock solution in DMEM. The cells were stained directly in their culture chamber for 40 min in an incubator. Following incubation, the cells were imaged live. 

The red-to-green ratio was then analyzed after a background subtraction with theImageJ-win 32 software, using a quantitative analysis based on an intensity measurement of specific selected ROIs. 

### 4.7. Mitochondria Function Analysis

The mitochondrial morphologyof both 3D-HTMC and RA-SH-SY5Y cells, treated as above-mentioned, was monitored using MitoTracker dye (Life Technologies), a cationic fluorophore that is able to electrophoretically accumulate into the mitochondria. Briefly, 1 mM stock solution of the MitoTracker probe Deep Red FM (Invitrogen^TM^) was dissolved in DMSO and stored at −20 °C. A fresh staining solution (100 nM) was prepared each time before use by diluting the stock solution in DMEM. The cells were stained for 40 min in an incubator after being freed from Matrigel^TM^, according to the manufacturer’s instructions. Following incubation, the cells were imaged live. Confocal microscopy images were converted to grayscale, inverted to show mitochondria-specific fluorescence as black pixels, and the threshold was adjusted to optimally resolve individual mitochondria. Every single mitochondrion was then analyzed for morphological characteristics, such as area, perimeter, circularity [perimeter^2^/(4π × area)] and solidity. 

### 4.8. Human Apoptosis Antibody Array C1 

An analysis of the apoptosis proteomic profile of 3DHTMC and 3D RA-SH-SY5Y after treatments was carried out using thecommercial Human Apoptosis Antibody Array C1 (RayBio^®^; Norcross, GA, USA), a semi-quantitative detector of human proteins. 

Sample preparation: At the end of the experimental time, cells were freed from Matrigel^TM^ and lysed using RIPA Buffer (Sigma-Aldrich) in combination with protease inhibitor cocktail tablets (Roche Diagnostics GmbH, Germany). After 30 min at 4 °C, the samples were centrifuged in a microcentrifuge for 15 min at 14,000 rcf and 4 °C. The supernatant of each sample was placed in a fresh tube and kept on ice or stored at −80 °C. 

Apoptosis Array: Each antibody array chip was pre-incubated with a specific buffer (provided in the kit) for 30 min at room temperature before sample incubation. Then, after removing the specific buffer, 1 mL of original lysate sample atthe concentration of 2–10 mg/mL was added onto the antibody printed side of an array chip and incubated for 1.5 to 5 hat room temperature or overnight at 4 °C. At the end of the incubation period, each sample was removed and the array chips were repeatedly washed, according to the manufacturer’s instructions. Therefore, we proceeded with the Biotinylated Antibody Cocktail (provided in the kit) incubation for 1.5 to 5 h at room temperature or overnight at 4 °C. At the end of the incubation period, each array chip was washed as described above and incubated with 1X HRP-Streptovidin (provided in the kit) for 2 h at room temperature. After further washing, each array chip was incubated with detection buffer (provided in the kit) for 2 min. The array chips were then transferred to a dark room to transfer the chemiluminescence signals onto X-rays plates. 

The intensity of the protein array signals was analyzed using a BIORAD Geldoc 2000, and each protein spot was normalized against positive control spots printed on each membrane. The data analysis was conducted according to the protocol directions of the Human Apoptosis Array C1. 

### 4.9. Annexin V

The apoptosis-induced morphological changes in both 3DHTMC and 3D RA-SH-SY5Ycells after treatments were detected using Annexin V, FITC and PI apoptosis detection kit (Dojindo Molecular Technology, Inc., Rockville, MD, USA). While Annexin V, a calcium-dependent phospholipid-binding protein, is an early apoptosis marker due to it binding with phosphatidylserine, propidium iodide (PI) is considered a late-stage apoptosis marker due to it entering the cells once cell membrane integrity is lost. 

Briefly, cells were freed from Matrigel^®^, according to the manufacturer’s instructions. Then, the cell suspension was centrifuged at 1000 rpm for 3 min, removing the supernatant. The cells were then washed twice with PBS and centrifuged at 1000 rpm for a further 3 min. After removing the supernatant, the cells were re-suspended in 100 μL of 10-fold diluted Annexin V binding solution, to which 5 μL of Annexin V, FITC conjugate and 5 μL of PI solution were added. Finally, the apoptotic cells were identified by confocal microscopy after 15 min of incubation at room temperature. 

### 4.10. Confocal Analysis 

Undifferentiated SH-SY5Y andRA-SH-SY5Y were plated at a density of 200,000 cells/μ-dish 35 mm (Ibidi, Munich, Germany). After 24 h of standard culture conditions, cells were fixed in 4% paraformaldehyde and permeabilized with 0. 3% Triton X-100 (Sigma Aldrich, Germany), and then theRA-SH-SY5Y cells were incubated in PBS containing 5% of Bovine Serum Albumin (Sigma-Aldrich) and 1:200 βIII Tubulin (Sigma Aldrich) for 1 h at RT.

All fluorescence signals were captured with 60× oil-immersion objective N. A. 1.4 by a Leica TSC SP microscope (Leica Microsystem, Wetzlar, Germany) equipped with 476, 488, 514, 543 and 633 excitation lines. Signals from different fluorescent probes were taken in sequential scan settings.

### 4.11. Statistical Analysis

GraphPad Prism 7.0 (GraphPad. Software, San Diego, CA, USA) was employed for the statistical analysis. For intergroup comparisons of undifferentiated and differentiated RA-SH-SY5Y cells, the unpaired *t*-test was used, whereas for the viability assay, JC1 fluorochrome intensity, annexinV and MitoTracker, a two-way analysis of variance (ANOVA) was performed for single comparisons, followed by Bonferroni’s post-test for multiple comparisons.

## Figures and Tables

**Table 1:** Primer sequences used for real-time quantitative polymerase chain reaction analysis.

Gene	Gene Bank	Forward	Reverse
MAP2	NM_002374. 3	TGCCATCTTGGTGCCGA	CTTGACATTACCACCTCCAGGT
HPRT	NM_000194. 3	GGTCAGGCAGTATAATCCAAAG	TTCATTATAGTCAAGGGCATATCC

## Data Availability

This present article reports only original data that were not published elsewhere.
